# 

**Published:** 1907-06-15

**Authors:** 


### Dental Register

[illegible] JUL -1. 90[illegible]

**NELVILLE S. HOFF, Editor,**
**Ann Arbor, Mich.**

Vol. LXI — JUNE, 1907 — No. 6

**SAMUEL A. CROCKER & CO., Publishers**
**OHIO DENTAL AND SURGICAL DEPOT**
**35 W. FIFTH STREET, CINCINNATI, O.**

PRICE, ONE DOLLAR A YEAR

Entered at the Postoffice at Cincinnati O., as second-class mail matter.

